# Data-driven assessment of eQTL mapping methods

**DOI:** 10.1186/1471-2164-11-502

**Published:** 2010-09-17

**Authors:** Jacob J Michaelson, Rudi Alberts, Klaus Schughart, Andreas Beyer

**Affiliations:** 1 Cellular Networks and Systems Biology, Biotechnology Center - TU Dresden, Dresden, Germany; 2 Helmholtz Center for Infection Research, Braunschweig, Germany

## Abstract

**Background:**

The analysis of expression quantitative trait loci (eQTL) is a potentially powerful way to detect transcriptional regulatory relationships at the genomic scale. However, eQTL data sets often go underexploited because legacy QTL methods are used to map the relationship between the expression trait and genotype. Often these methods are inappropriate for complex traits such as gene expression, particularly in the case of epistasis.

**Results:**

Here we compare legacy QTL mapping methods with several modern multi-locus methods and evaluate their ability to produce eQTL that agree with independent external data in a systematic way. We found that the modern multi-locus methods (Random Forests, sparse partial least squares, lasso, and elastic net) clearly outperformed the legacy QTL methods (Haley-Knott regression and composite interval mapping) in terms of biological relevance of the mapped eQTL. In particular, we found that our new approach, based on Random Forests, showed superior performance among the multi-locus methods.

**Conclusions:**

Benchmarks based on the recapitulation of experimental findings provide valuable insight when selecting the appropriate eQTL mapping method. Our battery of tests suggests that Random Forests map eQTL that are more likely to be validated by independent data, when compared to competing multi-locus and legacy eQTL mapping methods.

## Background

For decades scientists have used a variety of analytical techniques to relate allelic inheritance patterns in the genome to variation in continuous physical traits of interest. The goal of such analyses is often to locate quantitative trait loci (QTL), or genomic locations that exert an influence on the manifested trait. Understanding the genomic location of these genetic control points may provide insight into the genetic and molecular framework responsible for enabling the trait.

In the past decade, the advent of the DNA microarray and other high-throughput molecular technologies has updated the paradigm of the QTL. A QTL where mRNA expression is the complex trait of interest is generally referred to as an expression QTL or eQTL [1]. By using DNA microarrays eQTL can be measured for basically all genes in the genome, rendering eQTL data information rich and potentially very powerful. eQTL have been studied in yeast, mouse, rat, human, and plants [2-6] and eQTL have proven to be useful for elucidating the molecular mechanisms of human diseases [7-10].

Although complex traits are by definition controlled by the coordination of multiple genes, the prevailing techniques for mapping them have been deeply rooted in univariate thinking - testing for genetic association to a trait one locus at a time, ignoring combinatorial effects and interactions. In contrast, Broman and Speed [11] defined the QTL problem as one of multivariate variable selection, where ideally all loci and their combinations are allowed to enter and exit the model as the data dictate. Viewing eQTL mapping as a variable selection problem opens the door to using a host of machine learning algorithms which have rarely, if at all, been applied to QTL and eQTL studies [12-15]. Such a fresh look at the QTL problem may help to uncover latent and meaningful information in otherwise underexploited data.

A systematic comparison of eQTL mapping approaches is necessary to inform the research community which methods work best and in which contexts. Toward that goal, the purpose of this work is twofold. First, we establish a framework for comparing available eQTL mapping methods based on the tendency of each method to map eQTL that are systematically supported by external biological data. This is important because methods papers proposing new (e)QTL mapping techniques often draw their conclusions either solely or largely on the basis of simulated data [11,12,14,16-21]. This is perhaps understandable in the case of earlier work with QTL, where only a limited number of phenotypes were available and external knowledge about their context and probable genetic regulators was not available in a systematic form, making biology-based benchmarking difficult. However, this is not the case in the era of eQTL. Although some genes remain uncharacterized, there are rich sources of data for many genes that give insight about their role and context within the cell. Such knowledge is often contained in databases like the Kyoto Encyclopedia of Genes and Genomes (KEGG) [22], which makes using it as a basis for a benchmark easier. Our battery of knowledge-driven benchmarks consists of 1) assessing the proportion of *cis*-eQTL recovered by each method, 2) testing each method's high-scoring eQTL for enrichment of loci related to the target by KEGG pathway information, and 3) agreement of each method's high-scoring eQTL with systematic loss-of-function studies. In this framework we tested three variable importance measures from Random Forests (RF) [23] as well as sparse partial least squares (SPLS) [12], the lasso [24], the elastic net [25], Haley-Knott regression (HK) [20], and composite interval mapping (CIM) [19]. We also performed simulations to complement the findings of the knowledge-driven benchmarking framework. We show that multi-locus methods in general (Random Forests, SPLS, lasso, elastic net) are better at recovering biologically meaningful loci than traditional QTL mapping methods such as HK and CIM. Second, we demonstrate that based on both simulations and the knowledge-driven benchmarks, RF shows superior performance as an eQTL mapping method. RF has previously been applied to genome-wide association studies (GWAS) and QTL studies [14,15,17,26,27]. The contribution of our work, however, lies in the discovery that the most naive measure of variable importance in RF, the variable selection frequency (RFSF), actually performs much better than the more popular permutation importance (RFPI) in this context. Since RFSF has been ignored in all previous works using RF in the QTL or GWAS context, its use here represents a novel eQTL mapping method with demonstrated superior performance.

## Results

In order to evaluate the performance of the eQTL mapping methods in a comprehensive way, we used both simulated data and a variety of published and previously unpublished experimental data from mouse and yeast. The mouse data sets include gene expression data from four tissues of recombinant inbred (RI) BXD mouse strains: regulatory T-cell (H. Chen, RA, and KS, unpublished data), lung (RA, L. Lu, R. Williams, and KS, unpublished data), hematopoietic stem cells [28], and hippocampus [29]. The yeast data were taken from [30]. Further details are available in the methods section.

We note here that one of the goals of this comparison is to determine how susceptible each method is to the effects of linkage disequilibrium. In light of this goal we used all genotype data as-is, without prefiltering or fusing markers, or assigning surrogate eQTL post-hoc. This enables a straightforward comparison across all mapping methods.

### Simulations

**Figure 1:**
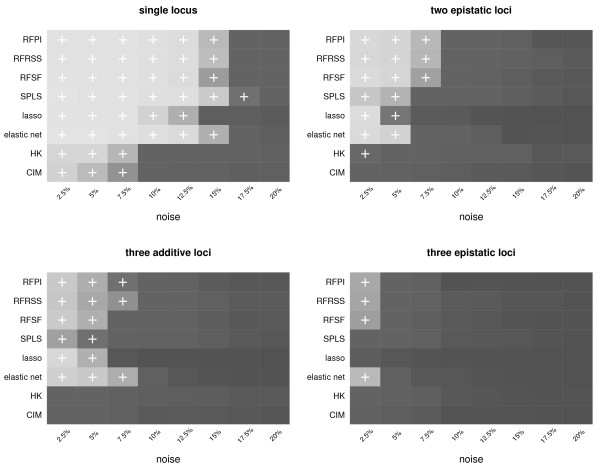
**Results of the simulated eQTL models**. Results of the simulated eQTL models. Each method-noise level combination where all of the causal loci were contained in the 99*^th ^*percentile of scores is marked with a '+'. Ranking within the 99*^th ^*percentile (of the worst-ranking of the causal loci) is indicated by the shade of gray, with lighter shades indicating better ranking.

We first set out to examine the performance of each method when the underlying model generating the data was known completely. We used the actual BXD genotypes and generated traits based on four models: single causal locus, two epistatic causal loci, three additive causal loci, and three epistatic loci. These configurations were sufficient to clearly distinguish the performance of the methods. Further details of the construction of the simulated data are given in the methods section. The goal of this investigation was to determine how well each method performed at placing *all *causal loci in the 99*^th ^*percentile of scores, over a range of increasing Gaussian noise in the trait. The results are given in Figure 1. In the single locus scenario, the performance gap between the newer multi-locus methods (RF, SPLS, the lasso, and the elastic net) and the legacy methods (HK and CIM) is quite apparent. In the single locus case, HK and CIM are unable to correctly identify causal loci in traits with more than 7.5% noise, and fail almost completely at pinpointing causal loci in the more complex two and three locus models. The elastic net and RF deliver comparable performance in the more complex models, with RF performing better in epistatic scenarios and the elastic net performing slightly better in the three-locus additive model. It should be noted that while SPLS, the lasso, and the elastic net do not explicitly search for interactions, they may still find loci participating in epistasis due to small but detectable marginal effects of the interaction.

### cis-eQTL counts

**Figure 2:**
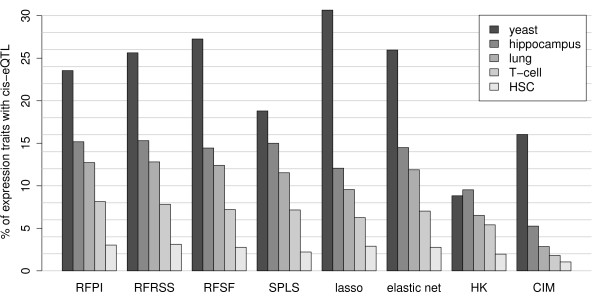
**Percentage of expression traits with a recovered cis-eQTL**. For each experimental data set, we calculated the percentage of transcripts which had a marker scoring in the 99*^th ^*percentile that co-localized with the genomic location of the target gene.

A "back of the envelope" approach for gauging the practical performance of a mapping method is the proportion of *cis*-eQTL found among all target transcripts in experimental data. Since promoter regions are often polymorphic, one would expect under optimal conditions to be able to recover an eQTL at the genomic location of many of the examined target transcripts. In this sense, the "external information" used in the benchmark is the knowledge of the genomic location of the gene -- which, when compared to QTL in general, is information unique to eQTL. The results of this assessment are shown in Figure 2. Taken individually, no single method dominated the others. However, the legacy methods (HK and CIM) again showed poor performance when compared to their more modern counterparts. A relationship between study size and proportion of recovered *cis*-eQTL is also uncovered, with the larger studies (yeast, mouse hippocampus and mouse lung with 114, 67, and 44 observations, respectively) generally yielding higher proportions of *cis*-eQTL than smaller studies (mouse regulatory T-cell and mouse hematopoietic stem cell with 33 and 22 observations, respectively).

### KEGG enrichment

**Figure 3:**
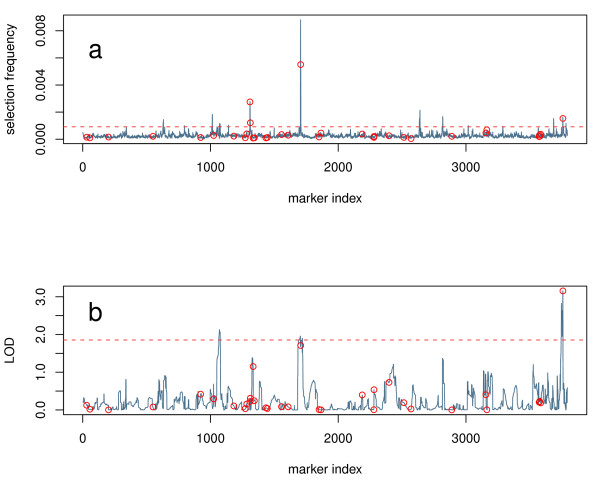
**Comparison of eQTL profiles**. An example eQTL profile for microarray probe set 1426838_at (Pold3) from the hippocampus data set, using RFSF (A) as the importance measure. Loci near genes participating in the same pathway (DNA replication) as the target gene (Pold3 - a DNA polymerase) are marked with circles. The 99*^th ^*percentile of the values in this profile is marked with a dashed line. (B) The same target probe set, using HK as the eQTL mapping method. The traditional mapping methods based on the LOD score tend to have very broad, blunt peaks, sometimes spanning most of a chromosome. Random Forests, on the other hand, produces very sharp, narrow peaks.

We used the pathway information available in the KEGG database to establish relationships between target genes and potential regulators. KEGG was chosen because of its position as a standard in pathway information and because it is generally a better reflection of the molecular relationships between genes (compared to GO for instance). However, in principle other sources of pathway information could be used. One would not expect to recover an entire pathway in every eQTL map, but on a large scale there should be some overlap between the eQTL and the relationships contained in KEGG. We assert that methods that show higher agreement with the information in KEGG are more desirable for eQTL mapping. We formalize this by assessing the enrichment of high-scoring eQTL for loci near genes known to participate in the same pathways as the gene whose expression trait is being mapped. A graphical depiction of this idea is given in Figure 3 and further details on the enrichment test are given in the methods section.

**Figure 4:**
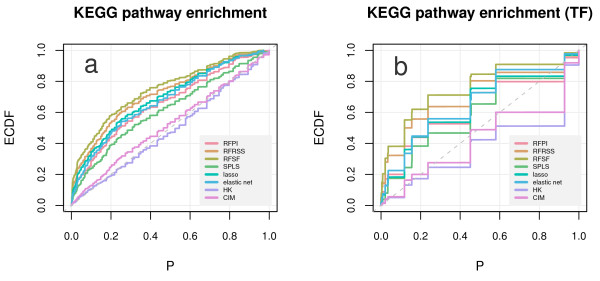
**Empirical cumulative distribution functions (ECDF) of enrichment *P *values**. The *P *values show the degree of enrichment among high-scoring yeast eQTL for genes that map to the same KEGG pathway as the target gene (A) and genes that map to the same pathway as the known transcription factors for the target gene (B). In both scenarios RFSF achieved the best performance in recovering loci enriched for pathway-related genes.

We tested pathway enrichment in yeast and mouse eQTL separately. For yeast, we included an additional enrichment test, which connected target genes not to pathways in which they participate, but to pathways in which the target's known transcription factors participate. We used the distributional properties of the enrichment *P *values to compare the eQTL mapping methods, with results for the yeast data shown in Figure 4. It should be noted that HK did not deviate significantly from the uniform distribution in either the pathway member or the TF-centric enrichment tests (*P *= 0.72 and *P *= 0.07, respectively, by the Kolmogorov-Smirnov test). In contrast, RFSF showed superior performance on the yeast data (*P *= 1.56 × 10^-133 ^and *P *< 10^-324 ^for the pathway member and TF-centric KEGG enrichment tests, respectively).

**Figure 5:**
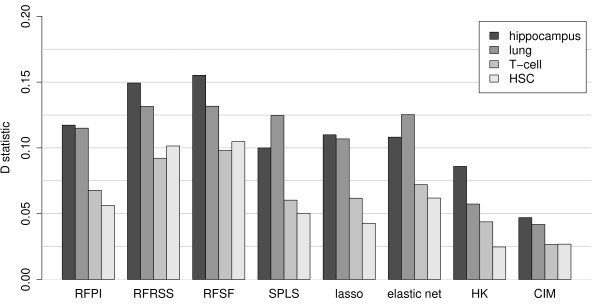
**Enrichment of KEGG pathway members in top-scoring loci in mouse tissues hippocampus, lung, regulatory T-cell, and hematopoietic stem cell**. The enrichment test procedure is the same as shown in Figure 4, but here the performance is summarized as the D statistic (maximum deviation from the uniform distribution) obtained from the Kolmogorov-Smirnov test.

The mouse data showed more modest enrichment across all tissues and with all methods, suggesting perhaps that larger studies are needed to better recover the complex regulatory systems present in higher eukaryotes (Figure 5). All methods yielded significant deviation from the uniform distribution in each tissue (P < 0.05 by the KS test). Again, RFSF yielded the greatest degree of enrichment in all tissues. SPLS, the lasso, and the elastic net produce sparse models, which means that not all loci are assigned a coefficient as a score. This had the effect that for a small minority of expression traits, the 99*^th ^*percentile of scores contained a small number loci with scores of 0. We examined whether this effect put these sparse methods at a disadvantage for the enrichment tests. We found no systematic relationship between enrichment *P *value and the number of 0 scores in the 99*^th ^*percentile.

### Mutant expression change enrichment

Finally, we combined data from two systematic loss of function studies [31,32] to see which method produced eQTL that agreed most with the mutant data.

In this test, we collected the maximum absolute expression change observed for each target gene when genes co-localized with eQTL in the 99*^th ^*percentile are mutated. These values were aggregated over all target genes, forming a distribution for each eQTL mapping method. We compared these distributions to a null distribution (see methods for details) via the Kolmogorov-Smirnov test. We assert that the method that yields eQTL that are enriched for large changes in expression in the mutant study is the most desirable method.

**Figure 6:**
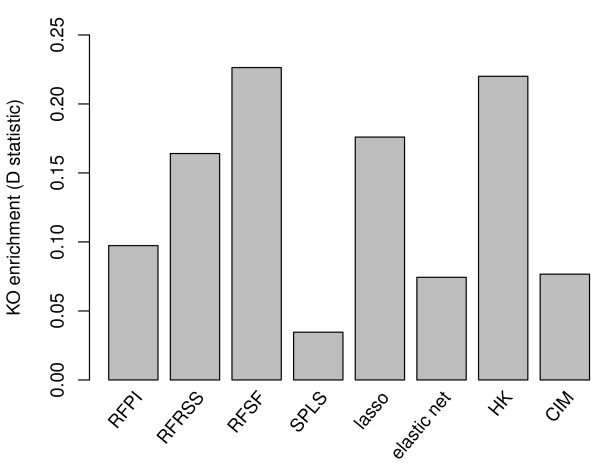
**Enrichment of high-scoring eQTL for mutant expression changes**. We used large-scale loss-of-function gene expression studies in yeast to determine whether high-scoring eQTL were near genes that, when mutated, perturbed the expression of the target gene. All methods showed significant enrichment for eQTL causing large expression changes when genes proximal to the eQTL are mutated, though the degree of enrichment varied widely. RFSF showed the most significant enrichment with *P *= 1.03 × 10^-99^.

All methods produced score distributions that deviated significantly from the null distribution, suggesting that there is indeed consistency between the yeast eQTL data and independent mutant data. Although all methods showed significant deviation from the null, the magnitude of enrichment varied widely (Figure 6). RFSF showed the most significant enrichment, followed closely by HK.

## Discussion

### High-throughput data make functional benchmarking of eQTL mapping methods possible

Augmenting eQTL with independent information has been done previously to strengthen hypotheses suggested by the eQTL data [33-36]. Although these applications demonstrate a certain degree of correspondence between eQTL data and external data sources, and imply that such correspondence is desirable in an eQTL mapping method, no benchmarks based on the systematic recovery of biological information have been proposed and applied to a wide variety of mapping methods and data sets. Validating the performance of mapping methods is important not only for those whose analysis ends with an eQTL map, but also for more sophisticated algorithms such as Lirnet [37] and Geronemo [38] which build on top of basic mapping concepts. Our analysis, combined with previously cited works that integrate eQTL with other data, show that there is indeed agreement among eQTL and data from different sources. Maximizing this agreement should be a core objective of future mapping techniques. We hope that this approach to benchmarking, in addition to traditional simulated benchmarks, will help practitioners find the appropriate method now, and lead to the development of better mapping methods in the future.

### Multi-locus eQTL mapping methods outperform legacy methods

With few exceptions, the legacy methods -- HK and CIM -- stood out as the poor performers, particularly in the simulations, *cis*-eQTL proportions, and enrichment for KEGG pathway relationships. In preliminary analyses, we found related univariate mapping methods such as EM interval mapping [21] and ANOVA to have performance almost indistinguishable from HK (data not shown). This observation is important because even at the time of this writing there are still eQTL papers being published that use legacy mapping methods for their analysis [39-42], ostensibly because the more modern methods are not as accessible. In light of our results, we expect that these studies have not exploited the full potential of the collected data. This represents a challenge for the computational community of working to promote not just the development, but also the adoption of these more advanced methods.

There is a fundamental difference in how the legacy linear methods (HK, CIM) and the multi-locus linear methods (SPLS, lasso, elastic net) score loci. The univariate mapping methods rely on a LOD score (or a *P *value in the case of one-way ANOVA) that expresses the significance of the estimated correlation between a single marker and the trait, resulting in thousands of individual modeling attempts per expression trait. The multi-locus methods, in contrast, assign coefficients to multiple loci in a single final model. These coefficients are then used as locus scores. The disparity in performance between the two classes of methods is likely a result of scoring by contribution to the model (multi-locus approach), rather than scoring by significance (univariate approach).

RF offers a third paradigm for scoring that is conceptually similar to the coefficient approach of the multi-locus linear methods, though distinct in implementation. Each of the three importance measures derived from RF measures a locus' average contribution in an ensemble of models. This differs from the coefficient approach in that it is a summary of multiple models, each including multiple loci, rather than a summary of a single model including multiple loci. Additionally, the multi-locus linear methods do not implicitly allow for the inclusion of epistatic interactions in the locus scoring process, while RF does.

It should be noted that the benchmarking process described in this work did not focus on the methods' abilities for statistical inference, that is, determining whether a locus *significantly *explains an expression trait. Instead, our benchmarks focused on which methods prioritized the loci with the greatest degree of effectiveness over a large panel of data. If statistical inference is desired, appropriate permutation of the data can be performed to obtain a null distribution of scores for the chosen method, which can then be used to assess significance of the scores.

**Figure 7:**
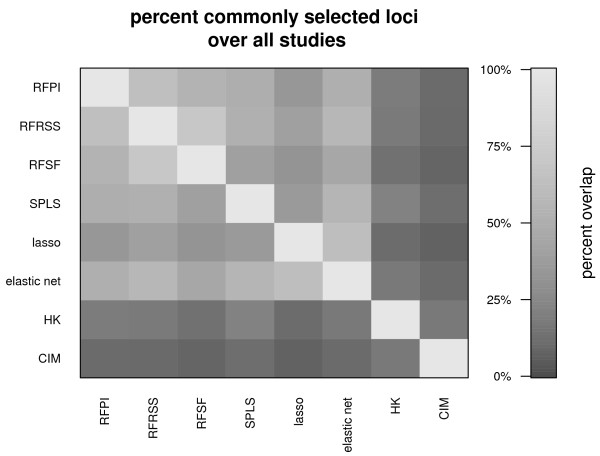
**Agreement between methods expressed as the overlap of selected loci, over all experimental data sets**. In general, the multi-locus approaches showed much more consistency with each other. The average percent overlap among RFPI, RFRSS, RFSF, SPLS, lasso, and elastic net was 49% (ranging from 31% to 67%), while HK and CIM had 17% of top loci in common.

We evaluated all experimental data sets and compared the loci that each method scored in the 99*^th ^*percentile. In general, the multi-locus approaches showed agreement amongst themselves, with an average 49% overlap. Figure 7 highlights the lack of consistency between the legacy methods and the multi-locus methods, and amongst themselves.

### Random Forests selection frequency maps the most biologically consistent eQTL

Random Forests (RF) [23] is a classification and regression algorithm based on fitting an ensemble of trees. When mapping eQTL, RF fits decision trees by using markers as predictor variables, i.e., each node in a tree corresponds to a split of the population based on the genotype at the selected marker. By combining an ensemble of many diverse decision trees, RF guards against overfitting and also provides several measures of predictor variable importance. In this work, these measures of variable importance are used to map eQTL.

**Figure 8:**
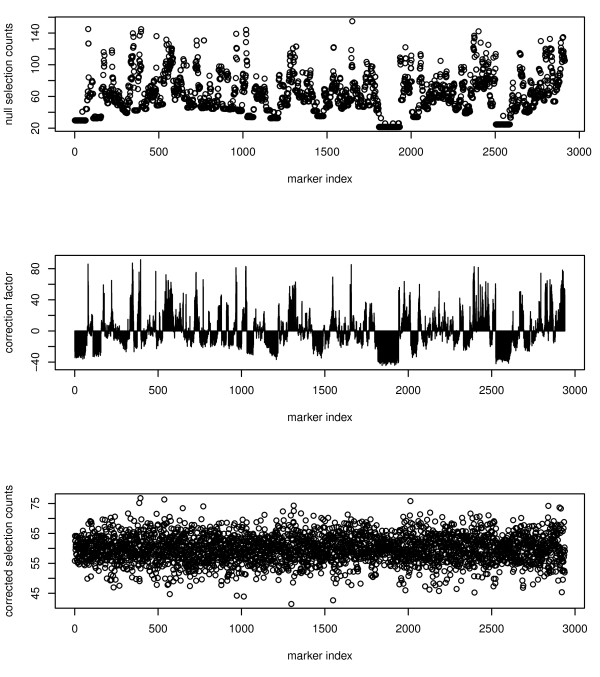
**Bias estimation and correction in RFSF**. Under the null hypothesis (no association between trait and genotypes), RFSF is biased towards variables with low correlation to others (top panel). The bias is estimated by fitting a forest to Gaussian noise, and a correction factor is derived by determining how much more or less frequently a marker is selected than the mean (middle panel). By subtracting the correction factor from the observed RFSF, the selection bias is removed (compare top panel to bottom panel).

Although multi-locus methods in general outperformed the legacy methods HK and CIM, RFSF showed the most consistent performance overall. In the simulations and *cis*-eQTL proportion test it was among the best, and in the KEGG and mutant enrichment tests it outperformed the competitors. This finding is somewhat surprising because RFSF is virtually ignored as a variable importance measure in most applications of RF, including QTL and GWAS [14,15,17,26,27]. Avoiding RFSF may have several explanations. For instance, it has been shown previously that RFSF can be biased. This bias manifests itself in the case of continuous or categorical predictors that vary widely in their scales or number of categories [43]. This is typically not an issue in the case of genotype data, where all predictors are categorical with the same number of categories. However, RFSF can also be biased when there is a significant degree of correlation between predictors, which is the case with genotype data. Under these conditions, RFSF preferentially selects variables (markers) with low correlation to other variables; markers in linkage disequilibrium are under-selected. In order to estimate and account for this bias, we add or subtract the deviation from the mean selection frequency observed under the null hypothesis (no association between trait and genotype data). See methods and Figure 8 for details.

**Figure 9:**
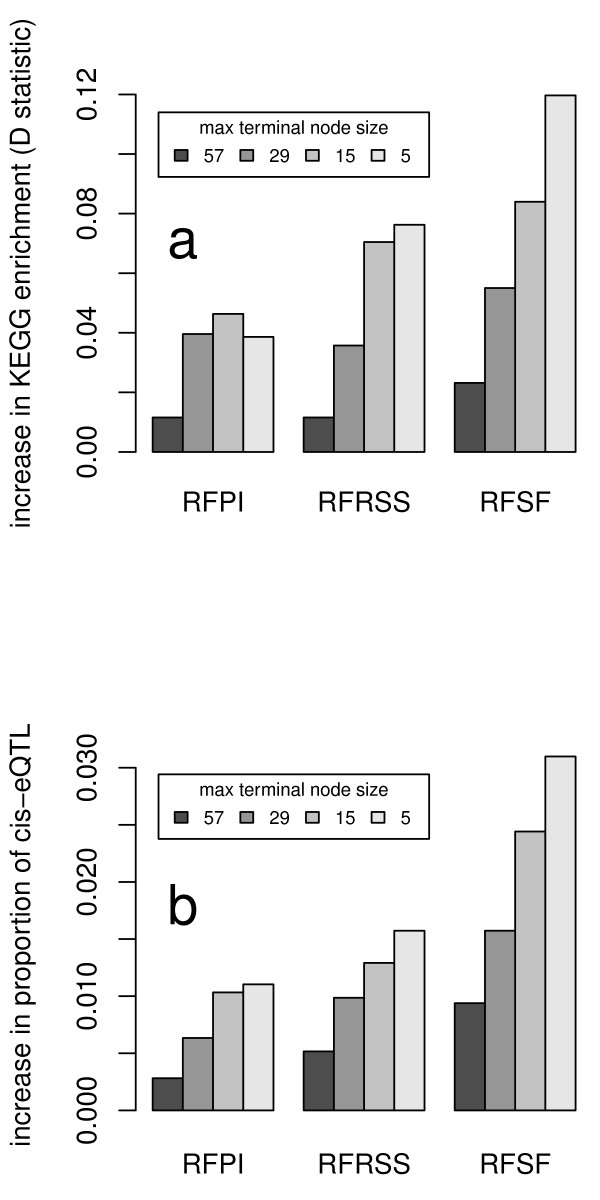
**Effect of varying Random Forests tree depth on performance**. The effect of varying Random Forests tree depth on performance as measured by the distributional deviation of the enrichment *P *values from the uniform distribution (A) and the percentage of expression traits with a *cis*-eQTL (B). Smaller node sizes correspond to deeper trees. The permutation importance and RSS importance improve modestly with deeper trees, whereas selection frequency shows more marked improvement with deeper trees. The improvement is measured with respect to forests that stop after the root split (nodesize 114).

**Figure 10:**
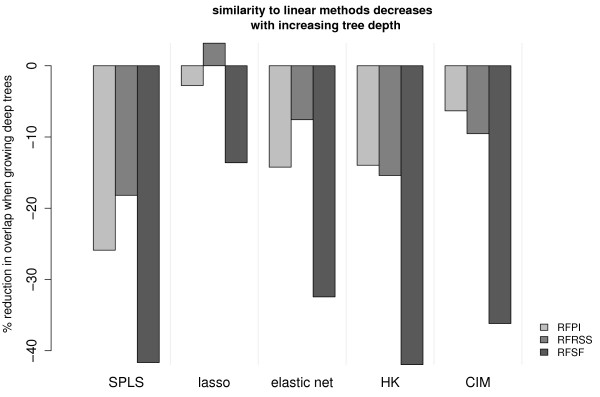
**Overlap of RF and linear methods while increasing RF tree depth**. In general, deeper trees caused the RF importance measures to diverge from the linear methods in terms of which loci were given the top scores. The effect is particularly pronounced for RF selection frequency (RFSF).

We decided to investigate further the potential reasons why RFSF performed better than the more typically used RFPI or RFRSS. We hypothesized that perhaps RFSF picked up on smaller effects near the leaves of the trees, i.e. it is able to detect loci with very subtle effects on the trait. To demonstrate this, we use the largest data set (yeast) and grew several RFs with different characteristic tree depths. We then tested these forests with the *cis*-eQTL proportion test and the KEGG enrichment test (see methods for details). We found that increasing the depth of the trees had a modest effect on the performance of RFPI and RFRSS, with an increase in percentage of *cis*-eQTL from 22.4% to 23.5% and 24.1% to 25.6%, respectively, and an increase in D statistic (for the KEGG enrichment test) from 0.186 to 0.225 and 0.241 to 0.318, respectively. Conversely, RFSF benefited more from the deeper forests, with an increase in percentage of *cis*-eQTL from 24.3% to 27.4% and an increase in D statistic (for the KEGG enrichment test) from 0.241 to 0.361 (Figure 9). In addition, we found that agreement with the linear methods (SPLS, lasso, elastic net, HK, and CIM) was at its highest when the tree growth was stopped early; similarity decreased with increasing tree depth. This effect was more pronounced for RFSF than for the other RF importance measures, which further suggests that the effects found near the leaves of the trees are connected to RFSF's superior performance (Figure 10).

To further explore this idea, we performed simulations where the expression trait was a function of eight loci, two with strong effects, and six with small effects. As expected, the loci with the stronger effects were used in splits closer to the root node. The causal loci with weaker effects were used to split closer to the leaves. In these simulations, RFSF scored the weak causal loci in the 99*^th ^*percentile 18.3% of the time, while RFPI scored the same loci in the 99*^th ^*percentile only 10% of the time. These simulations also showed that RFPI is tightly coupled to a variable's proximity to the root node, while RFSF can give high scores even if the variable is not used close to the root node.

From these investigations we conclude that RFPI and RFRSS both essentially determine variable importance near the roots of the trees, and that biologically important splits further down the tree are not adequately reflected in the overall importance scores. RFSF on the other hand, recovers more biologically meaningful predictor variables (loci) when trees are grown deep, suggesting that even splits far down the tree can be reflected in this importance measure. Epistatic effects are an example of where this phenomenon is important -- often genetic interactions are weak and only present in a subset of the population. Such conditional effects are likely to manifest themselves deeper in the trees. RFSF is an attractive measure in these situations.

Because of its demonstrated performance advantages in finding biologically relevant loci, its ability to implicitly consider epistatic interactions, as well as its straightforward and readily available implementation, we recommend using Random Forests for eQTL mapping. We have prepared a short tutorial and example R code demonstrating mapping eQTL with the bias-corrected selection frequency at http://cellnet.biotec.tu-dresden.de/RFSF.

### Marker density and analysis strategy

**Figure 11:**
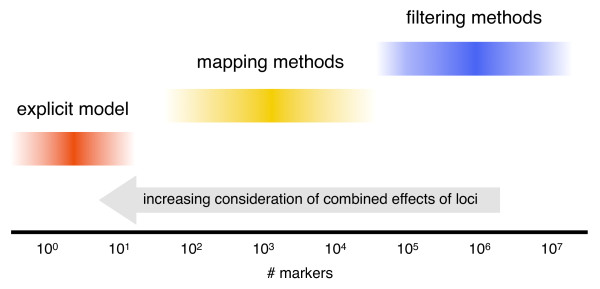
**Relationship between SNP density and analysis strategy for eQTL data**. The current state of computer hardware allows little if any consideration of joint effects of markers when millions of SNPs are considered for tens of thousands of expression traits. Simple univariate tests or expert knowledge are often employed to reduce the number of considered SNPs to a range where mapping methods may be used and increased attention may be given to the interplay between loci. In the optimal case, successful application of mapping methods in many populations will yield an explicit model of the expression trait in terms of a smaller number of genetic loci, optionally including environmental effects.

In this work we examined studies with genotype data in the range of thousands of markers. With the advent of next-generation sequencing and other ultra high-throughput methods, we expect to see more and more studies with hundreds of thousands, millions, or even tens of millions of SNPs. We wish to put the presented work in context by drawing a distinction between filtering methods, mapping methods, and explicit models (Figure 11).

The state of computer hardware at the time of this writing makes the multi-locus methods presented here impractical for exhaustive evaluations of data sets with millions of SNPs and tens of thousands of expression traits. The current solution to this problem is to filter the SNPs to a more tractable number using univariate tests or expert knowledge [44-46]. Considering the joint effects of markers at this point is generally a fruitless effort, given the astronomical number of potential combinations and the problem of dealing with false positives.

As the number of markers considered falls into the tens of thousands, the problem transitions from filtering to mapping. Mapping is a combination of modeling and feature selection, and the methods we explored in this work address the mapping problem. Here the interplay between loci becomes important for accurately identifying the causal regions that should be included in an explicit model of the trait.

Once causal loci have been identified reliably and the relationships between them have been characterized (additive vs. dominant, epistatic vs. additive, etc.), one can construct a linear model, usually consisting of a handful of terms, that accurately describes the trait as a function of the genetic state of the organism. Such an explicit model, though desirable, is rarely attained.

### Implications for related mapping problems

Most of the conclusions from our work have implications beyond eQTL mapping. Ideally, the concept of a knowledge-driven benchmark could be used for any physiological trait, but our approach depends on a fairly detailed knowledge of the molecular mechanisms underlying the mapped trait. Neither our notion of measuring the enrichment of regulator-target gene groups in common pathways, nor our counting of *cis*-eQTL is immediately extendible to physiological traits. Still, taken together, the evidence from this study indicates that QTL mapping - whatever the trait - should be performed using a multi-locus method. Using univariate methods such as HK will lead to severe underexploitation of the data.

Some of the more specific conclusions from our work will need further validation in other organisms and populations. For example, the study populations used here all had roughly a 50/50 distribution of two possible alleles at each marker. Human populations are characterized by very uneven distributions of SNPs, where minor alleles can be extremely rare in a given population. Such a change in the characteristics of the data could influence the ranking of the individual methods. However, such fluctuations in the individual rankings are still unlikely to affect the general conclusion that multi-locus methods produce more informative results than univariate methods, even in GWAS and linkage studies in outbred populations [47-51].

**Figure 12:**
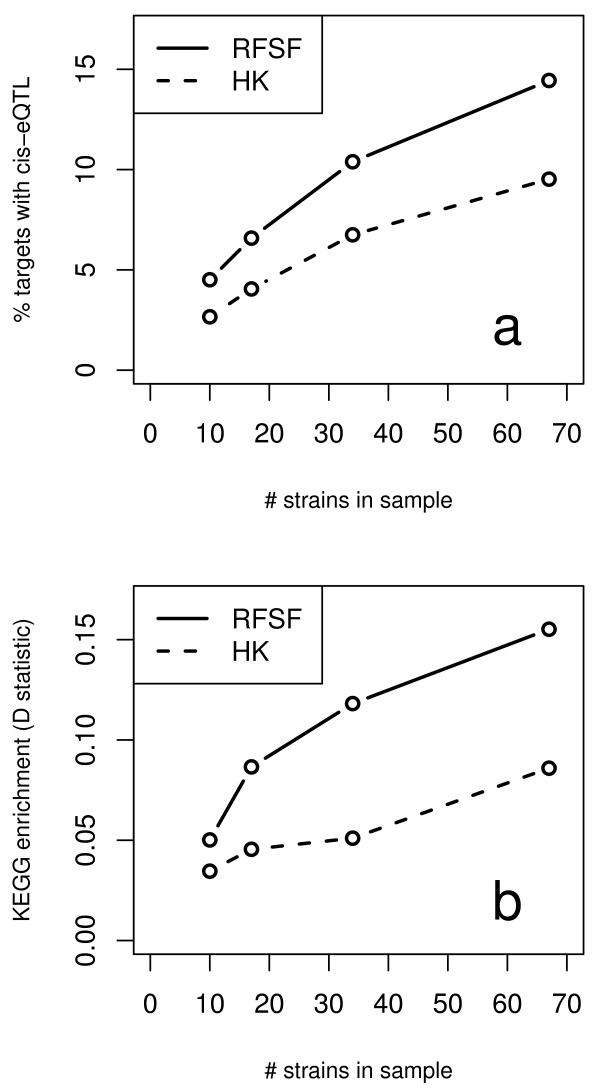
**Relationship between sample size and ability to recover biologically relevant loci**. Subsets of decreasing size (67, 34, 17, and 10 strains) were taken from the hippocampus eQTL study and eQTL were mapped using RFSF and HK. Performance was evaluated with the *cis*-eQTL and KEGG enrichment benchmarks. Both RFSF and HK improved performance when additional strains were added, though the performance RFSF was consistently better than HK in both benchmarks for all sample sizes.

Finally, in this work we observed the expected relationship between study size and power to detect biologically interesting loci. We explored this phenomenon explicitly by taking subsets of decreasing sample size from the hippocampus study, and then comparing two representative methods - here RFSF and HK - using the *cis*-eQTL and KEGG enrichment benchmarks. The results are depicted in Figure 12 and clearly show that while both methods show improvements with additional samples, it is RFSF, the multi-locus method, that shows consistently better performance, regardless of the sample size. This suggests that even in studies with small sample sizes, multi-locus approaches are preferable to single-locus methods.

## Conclusions

We have compared modern machine learning and regression eQTL mapping methods with more classical mapping approaches from statistical genetics, and evaluated the methods based on their ability to lead users to loci that are more readily supported by external information. We found that the modern methods, which freely allow the consideration of all loci simultaneously, generally outperform their classical counterparts in this regard. In particular, we found that Random Forests consistently mapped the most promising eQTL. Random Forests bias-corrected selection frequency, a novel importance measure, performed better in these tasks than the established permutation importance and RSS importance.

## Methods

### eQTL mapping

We used expression data from four eQTL studies in four different tissues in recombinant inbred BXD mouse strains: regulatory T-cell, lung, hematopoietic stem cells [28], and hippocampus [29]. We used only probe sets that mapped unambiguously to Ensembl gene IDs with KEGG annotations [22]. This resulted in a set of 6,121 probe sets for studies using the Affymetrix Mouse 430 2.0 array (lung, regulatory T-cell, and hippocampus) and 3,051 probe sets for the hematopoietic stem cell study, which used the Affymetrix U74Av2 array. Genotype data for the BXD recombinant inbred strains of mice used in these studies consisted of 3,794 markers and was downloaded from the GeneNetwork database [52]. In addition to the mouse data, we used the yeast eQTL study previously published in [30]. After filtering out probes with missing or otherwise ambiguous data, we were left with 4,501 gene expression measurements and 2,914 markers.

#### Random Forests

We used the reference implementation of Random Forests [53] in R for all mapping discussed in this work. We grew forests with 5,000 trees, the mtry parameter was set to the default (one third of the total number of markers) and the node size was also the default of 5, unless otherwise noted. We then extracted unscaled permutation importance measures (RFPI), residual sums of squares importance measures (RFRSS), and selection frequencies (RFSF) from the forests for use as the scores for each marker.

We estimated and accounted for bias in RFSF as follows. Using the actual genotype data as predictors, we fit 500 10-tree forests to independent draws from Gaussian noise. This resulted in 5,000 trees, equal in size to the forests used in this work. We collected the selection frequencies for each marker and subtracted the mean selection frequency to yield a vector of correction factors -- one value for each marker. Subtracting this vector of correction factors from the observed selection frequencies (from the observed data) gives bias-corrected selection frequencies (Figure 8). In the context of results in this work, all references to RFSF imply the bias-corrected RFSF, as described here.

#### Sparse partial least squares

Chun and Keles [12] recently introduced a method of eQTL mapping using sparse partial least squares, which included an R package and a thorough tutorial available online. We used the spls R package to map eQTL, performing cross-validation on every target to determine the optimal parameters for each fit. eta, the thresholding value, was allowed to vary between 0.3 and 0.7, to prevent both overfitting and a model that was too sparse to score multiple loci. The number of hidden components was allowed to vary from 1 to 5. A final fit was performed with the optimal parameters, and the absolute value of the coefficients was used as the score for each marker.

#### The lasso

The lasso [24], a regression shrinkage method, has previously been applied to QTL mapping [54], but to our knowledge has never been tested against competing mapping methods in the context of an eQTL study. For this work, we used the lasso as implemented in the elasticnet package for R. The lasso is a special case of the elastic net with lambda equal to (or very near) 0. For each target gene examined, we took the absolute value of the lasso coefficients for a fit performed with the s parameter determined by 10-fold cross-validation, with an imposed minimum of 0.5. These coefficients were used as the score for each marker.

#### The elastic net

The use of the elastic net [25] was the same as above for the lasso, except that lambda was set to 1. We found this value of lambda to be optimal after testing a sample of target genes over a range of lambda values (0.5,1,10,100).

#### Haley-Knott regression

We used the implementation of Haley-Knott regression [20] available in the qtl package for R. LOD scores were calculated at the marker locations.

#### Composite interval mapping

To perform composite interval mapping [19] we used the implementation in the qtl package for R, with the method argument set to "EM", and all other arguments set to their default. LOD scores were calculated at the marker locations.

### Simulations

To simulate eQTL with known underlying models, we used the full BXD genotype matrix, available from the GeneNetwork [52]. This matrix consists of 89 strains and 3,794 markers. Using this genotype data, we randomly selected one, two, or three markers (depending on the model to be simulated), and then simulated a trait by using a linear combination of the markers directly, or of logical operations on the markers (in the case of epistasis). All traits started with a baseline value of 9, before adding in the genetic effects. Genetic effects were added as follows: in the single locus model, a single marker was selected at random, and its vector of genotypes (where 1 = BB and 0 = DD) was multiplied by a coefficient, in this case 1. For the two-locus epistatic model, two marker vectors were selected at random, with each being multiplied by 0.25 and then summed. The epistatic component was added by applying the AND logical operation to the genotype vectors (where a 1 is a TRUE and a 0 is a FALSE) and then multiplying the result by a coefficient, in this case 1, and then adding to the additive component. Three locus additive and epistatic traits were constructed in a similar fashion. Gaussian noise with mean 0 was then added to the traits, over 8 levels of increasing standard deviation, which corresponded to 2.5, 5, 7.5, 10, 12.5, 15, 17.5, and 20% of the trait mean.

Each model type (i.e. single locus, two locus epistatic, etc.) was simulated independently 50 times, and each mapping method was applied to the same data. For each simulation and for each mapping method, the maximum (i.e. worst) rank among the set of causal markers was recorded in each noise level. The median of these values (over the 50 simulations) was used to reflect the performance of a given mapping method over the increasing levels of noise. Lower values represent the ability of a method to assign high scores to *all *causal loci.

### cis-eQTL counts

Performance based on the proportion of recovered *cis*-eQTL was assessed by counting the number of expression traits where a marker within 500 kb (for mouse) or 50 kb (for yeast) of the midpoint of the target gene's genomic location had a score in the 99*^th ^*percentile of the scores for the respective target gene. These cutoffs, though arbitrary, reflect the difference in complexity between the yeast and mouse genomes - the conclusions drawn from the benchmark are not heavily influenced by this choice. This number was then divided by the number of total expression traits examined for the respective data set.

### KEGG enrichment

Each expression trait we tested mapped to at least one KEGG pathway, and each gene found in the KEGG pathway was mapped to the nearest marker. If no marker fell within 5 Mb of a gene, the gene was omitted. For each expression trait, the markers having scores in the 99*^th ^*percentile were selected for the enrichment test. The hypergeometric test was used to test this set for the enrichment of markers mapping to genes participating in the same KEGG pathway as the target gene. If multiple pathways existed for any expression trait, all were tested and the minimum *P *value was used as the representative *P *value.

In the case of the yeast eQTL data, we additionally assessed enrichment of pathways in which transcription factors binding to the target gene participate. As a basis for mapping transcription factors to their targets, we used [55]. We did not attempt this test with the mouse data because of the lack of dense and reliable TF-target data for mouse.

Since in this test even randomly selected markers yield *P *values that deviate somewhat from the uniform distribution, we calculated an empirical null distribution of *P *values. To construct this distribution, we assigned scores to the markers, drawn randomly from a Gaussian distribution with mean 0 and standard deviation of 1. We then took the markers in the 99*^th ^*percentile and performed the proposed enrichment test. This was performed for an equivalent number of expression traits contained in the actual data sets. The actual enrichment *P *values were corrected against this empirical null distribution of enrichment *P *values.

We plotted the empirical cumulative distribution function (ECDF) of the corrected enrichment *P *values for each method. As a summary measure for each method's deviation from the uniform distribution, we used the D-statistic as given by the Kolmogorov-Smirnov test. The test was one-sided with the alternative hypothesis that the observed cumulative distribution function accumulated faster than the reference (i.e. uniform) distribution.

### Mutant expression change enrichment

Systematic loss of function data in yeast [31,32] was used to assess which eQTL mapping method tended to agree most with the regulatory relationships suggested by experimentally deactivating upstream regulators. We mapped each repressed gene to its nearest marker. Then, for each expression trait from the yeast eQTL study, we looked at markers in the 99*^th ^*percentile of scores for that target. For markers mapping to experimentally repressed regulator genes, we collected the maximum absolute log_2 _expression ratio (repressed expression divided by wild-type expression) for the appropriate target gene, aggregating them over the whole set of mapped expression traits. We then compared the distribution of the selected maximum absolute log_2 _ratios generated by each eQTL mapping method by the Kolmogorov-Smirnov (KS) test, collecting the associated *P *value and D statistic. As a reference distribution in the KS test, a null distribution was constructed by a similar aggregation of maximum absolute fold changes, only with the association between scores and markers randomized for each target gene. The test was one-sided with the alternative hypothesis that the observed cumulative distribution function accumulated slower than the reference distribution. Distributions with a tendency toward higher scores and deviating significantly from the reference distribution suggest an agreement between the eQTL and loss-of-function studies.

### Variation of tree depth

To assess the impact of tree depth on each RF importance measure, we used the yeast eQTL data and recomputed eQTL maps for all expression traits, varying the nodesize argument to 5, 15, 29, 57, and 114.

The nodesize argument dictates whether or not a node may be split -- if the number of observations in the node under consideration is greater than nodesize, the node may be split. Otherwise the node is not split and is marked as a terminal node. The default value of nodesize is 5 -- this is the value used in the main body of the study. By selecting a nodesize of 114 (the number of samples in the yeast study), we ensure that splitting stops after the first split. The other values are intermediate steps, each about half the size of the last. We then assessed the improvement in the enrichment of KEGG pathway members and proportion of cis-eQTL identified when growing the trees deeper, using the forest with nodesize 114 as the baseline.

## Authors' contributions

JM and AB conceived the benchmarks and analysis. JM performed the analysis and drafted the manuscript. RA assisted in the analysis. KS conceived and carried out the lung and T-cell studies. All authors reviewed the final manuscript.
